# Association between ultra-processed food consumption and cognitive performance in US older adults: a cross-sectional analysis of the NHANES 2011–2014

**DOI:** 10.1007/s00394-022-02911-1

**Published:** 2022-07-01

**Authors:** Barbara R Cardoso, Priscila Machado, Euridice Martinez Steele

**Affiliations:** 1 Department of Nutrition, Dietetics and Food, Monash University, 264 Ferntree Gully Road, Notting Hill, VIC 3168 Australia; 2 Institute for Physical Activity and Nutrition (IPAN), School of Exercise and Nutrition Sciences, Deakin University, VIC, Australia; 3 Departamento de Nutrição, Faculdade de Saúde Pública, Universidade de São Paulo, Av. Dr. Arnaldo, 715, São Paulo, 01246-907 Brazil; 4 Center for Epidemiological Studies in Health and Nutrition, University of São Paulo, São Paulo, Brazil

**Keywords:** Ultra-processed food, Diet, Cognitive decline, Dementia, Older adults

## Abstract

**Purpose:**

This study evaluated the association between ultra-processed food (UPF) consumption and cognitive performance among older US adults.

**Methods:**

This cross-sectional study assessed 3632 participants aged 60+ years from the National Health and Nutrition Examination Survey (NHANES) 2011–14. Cognitive performance was assessed using the Consortium to Establish a Registry for Alzheimer’s Disease (CERAD), Word Learning test, Animal Fluency test, and the Digit Symbol Substitution test (DSST). Dietary intake was assessed using two 24-h diet recalls. Food items were classified according to the NOVA system, a classification based on the nature, extent, and purpose of industrial food processing. Linear regression models were used to evaluate the association of dietary share of UPF (% of daily energy intake) (categorized as tertiles) and cognitive test scores, adjusting for socio-demographic variables, physical activity, smoking status, and chronic diseases (cardiovascular diseases, diabetes, and depression). Models excluding participants with pre-existing diseases were carried out to address potential reverse causality.

**Results:**

On average, UPF accounted for 53% of total energy intake, ranging from 33 to 70% across extreme tertiles. Inverted U-shape association between UPF consumption and Animal fluency and DSST was observed. No significant associations were observed between the UPF intake tertiles and the cognitive test results. Nonetheless, UPF consumption was significantly associated with worse performance in Animal Fluency in older adults without pre-existing diseases (*P* < 0.05).

**Conclusion:**

UPF consumption was associated with worse performance in Animal Fluency among older people without pre-existing diseases. Decreasing UPF consumption may be a way to improve impaired cognition among older adults.

**Supplementary Information:**

The online version contains supplementary material available at 10.1007/s00394-022-02911-1.

## Introduction

The number of people living with dementia is rising as life expectancy increases worldwide. Given the enormous burden that dementia causes to individuals, families, communities, systems of care, and society at large [1], identification of modifiable risk factors for dementia enables public health measures to prevent or delay the cases of dementia. It has been estimated that 40% of dementia cases could be prevented by controlling 12 modifiable risk factors, which include diet along with physical activity, education, smoking, exposure to air pollution, low social contact, traumatic brain injury, and management of chronic conditions such as diabetes, obesity, depression, hearing impairment, and cardiovascular diseases [2].

Research has recently shifted from identifying the role of specific nutrients in dementia to focussing on the potential synergetic effects of dietary patterns in mitigating age-associated cognitive decline, and thus protecting against dementia. In this regard, evidence from observational studies indicates that high adherence to the Mediterranean diet (MedDiet) [3, 4], Dietary Approaches to Stop Hypertension (DASH) [5] diet, and the Mediterranean-DASH Intervention for Neurodegenerative Delay (MIND) diet [6] is associated with better cognitive performance and a reduced risk of dementias and/or slower cognitive decline rates [7, 8].

The important role of the extent and purpose of the industrial processing of foods belonging to these dietary patterns is being increasingly recognised, particularly the contribution of ultra-processed food (UPF). UPFs, according to NOVA classification system, are industrial formulations of processed food substances (oils, fats, sugars, starch, and protein isolates) that contain little or no whole food and typically include flavourings, colourings, emulsifiers, and other cosmetic additives [9]. UPFs are becoming dominant in diets globally and are replacing traditional diets based on unprocessed and minimally processed foods [10, 11]. In the US, average UPF consumption ranges from about 58% of total energy intake among individuals aged ≥ 60 years [12] to 67% among children and adolescents [13]. This is of great concern considering that UPF consumption has been associated with overall decline in the nutritional quality of diets [14], as well as with several chronic diseases such as overweight/obesity, metabolic syndrome, hypertension, diabetes, and cardiovascular diseases (CVD) [15–17]. Such associations were also observed in the US population [18–22].

Further to the association between UPF consumption and chronic diseases that increase the risk of age-associated cognitive decline, a recently published review speculated that a dysregulation of the microbiota caused by UPF consumption could also be a mechanism linking UPF and cognitive decline [23]. Considering these known risks associated with UPF consumption, we hypothesised that higher intakes of UPF are associated with worse cognitive performance in older adults. In this study, we investigated the association between UPF consumption and the performance in cognitive tests in a representative sample of older people in the US.

## Methods

### Study population

The National Health and Nutrition Examination Survey (NHANES) is a large, complex, multistage, survey of noninstitutionalised US population conducted by both the Center for Disease Control (CDC) and the National Center for Health Statistics to provide nationally representative estimates on the health and nutritional status [24]. This cross-sectional analysis included participants ≥ 60 years old from 2011–12 and 2013–14 cycles. The NHANES protocol was approved by the National Center for Health Statistics (NCHS) Research Ethics Review Board. All the NHANES participants provided with informed consent [25].

A total of 3632 participants ≥ 60 years old were assessed in the two NHANES cycles included in this analysis. Among them, 2934 individuals underwent cognitive assessment. Participants were further excluded due to missing data for dietary intake (221), leaving 2713 older people for analysis (Supplementary Fig. 1).

### Dietary assessment and estimated UPF intake

Dietary intake was assessed using two, non-consecutive 24-h dietary recalls. Data were collected by trained interviewers using the United States Department of Agriculture (USDA) Automated Multiple-Pass Method. The first dietary interview was conducted in person, followed by a second interview administered via phone 3–10 days later [24]. This study included participants who have reliably completed at least one of the dietary recalls. Dietary intake was reported as the average intake from both 24-h recalls when 2 days of data were available, and as day 1 otherwise.

In the dietary interviews, participants provided information on the type and amount of food and beverages they consumed in the previous day. The items recorded in the 24-h recalls were classified according to NOVA, a food classification based on the extent and purpose of industrial food processing, into four mutually exclusive groups: (1) unprocessed or minimally processed foods, (2) processed culinary ingredients, (3) processed foods, and (4) UPFs. Foods were classified into each of the four groups based on the variables “Main Food Description”, “Additional Food Description”, and “SR Code Description” from the NHANES 24-h recall datasets. Classification could be modified according to the variables “Combination Food Type” and “Source of food”. As such, most foods described as “Frozen meals” or “Lunchables”, as well as some items described as consumed in “Restaurant fast food/pizza” or acquired at a “Vending machine” were classified as UPFs. If an item was considered to be a hand-made recipe, the NOVA classification was applied to each of the underlying ingredients (Standard Reference Codes) as previously described [26]. For this study we used Food Codes energy values as provided by NHANES. For hand-made recipes, we calculated the underlying ingredient (Standard Reference Codes) energy values using variables from both the Food and Nutrient Database for Dietary Studies 6.0 [27] and 2013–2014 [28] and USDA National Nutrient Database for Standard Reference, release 26 [29] and 28 [30]. Classification was revised independently by two researchers and discrepancies were resolved by consensus. In this study, the exposure measure was the mean dietary contribution of UPF to total energy intake.

### Cognitive assessment

Trained interviewers administered the cognitive tests at the beginning of the face-to-face private interview. The NHANES uses the Consortium to Establish a Registry for Alzheimer’s Disease (CERAD) Word Learning test, the Animal Fluency test, and the Digit Symbol Substitution test (DSST) to assess different cognitive domains. The CERAD Word Learning test assesses immediate and delayed recall of new verbal information, a component of the memory domain; the Animal Fluency test evaluates categorical verbal fluency (executive function); the DSST assesses processing speed, sustained attention, and working memory. The two parts of the CERAD Word Learning test consist of (1) three consecutive learning trials, where the participant is requested to recall a list of ten unrelated words immediately after their presentation. Each word corresponds to one point, and the result is presented as a total score across the three trials (range 0–30); and (2) a delayed word recall test, performed after the two other cognitive tests. The result ranges from 0 to 10. For the Animal Fluency test, the participant is requested to name as many animals as possible within a 60-s time period. Each animal corresponds to 1 point and the result is presented as the total sum of points. For the DSST, the participant is presented a single sheet of paper where they are asked to match a list of nine symbols to numbers according to a key located on the top of the page. The task had 133 numbers and the participant had 2 min to complete it. The result is shown as the total number of correct matches. For all the tests, higher scores represent better cognitive function. All these tests have been validated in large epidemiological and clinical studies.

### Covariates

Demographics (age, gender, ethnicity, education, and poverty–income ratio), lifestyle information, and health history were collected by trained interviewers using a Computer-Assisted Personal Interviewing (CAPI) system. Ethnicity was categorized as Mexican American, Non-Hispanic white, Non-Hispanic black, or other/multiracial. Education attainment was categorized as incomplete high school, high school graduate, incomplete college, or college graduate. Poverty–income ratio, a measure that considers the ratio of household income to the poverty threshold after accounting for inflation and family size, was categorized into < 1.30 (poorer) or ≥ 1.30 (richer). Smoking status was categorized as smoker or non-smoker at the time of testing. Physical activity was assessed with a specific questionnaire (Global Physical Activity Questionnaire). Estimates of daily moderate and vigorous physical activity were calculated by multiplying the frequency per week by the duration (min) of physical activity divided by seven. *Z* scores for moderate/vigorous physical activity were then calculated using the mean and standard deviation of the NHANES ≥ 60 years sample. History of CVD was identified if the participant self-reported being informed by a physician about having congestive heart failure, coronary heart disease, angina pectoris, heart attack, stroke, high blood pressure, or high cholesterol levels as previously described [31]. Diabetes status was identified if the participant presented with glycohaemoglobin (Hb1Ac) ≥ 6.5% (Hb1Ac was measured in blood by the Tosoh Automated Hb1Ac Analyzer HLC-723G8) or if the participant self-reported being previously diagnosed with diabetes by a physician. Depression was identified with the Patient Health Questionnaire, a nine-item screening instrument that assesses the frequency of depression symptoms over the past 2 weeks. Four response categories were available for each question: "not at all", "several days", "more than half the days", and "nearly every day". The answers were assigned a point ranging from 0 to 3, and the final result corresponded to the sum of the points (range 0–27). Depression was defined as ≥ 10 points [32]. Participants underwent measurement of height and weight by trained interviewers to calculate body mass index (BMI). BMI was calculated as weight (kg) divided by squared height (m^2^), and then rounded to one decimal place. Participants were categorized according to BMI as follows: underweight (< 18.5 kg/m^2^), normal weight (18.5–24.9 kg/m^2^), overweight (25–29.9 kg/m^2^), and obese (≥ 30 kg/m^2^) as per the CDC cut-offs.

### Statistical analysis

Demographic and clinical characteristics were presented as mean with standard error (SE) for continuous variables, or % weighted (SE) for categorical variables. Data were compared across the tertiles of the dietary contribution of UPF (% of total energy intake; %kcal UPF) using linear regression models for continuous variables or Pearson’s chi-square for categorical variables.

We used the restricted cubic spline in the unadjusted regression models (Model 1) with five knots (5th, 27.5th, 50th, 72.5th, and 95th) as per Harrell’s recommendations [33] to examine the shape of the dose–response relationship curve between %kcal UPF (as a continuous variable) and cognitive test scores.

Cognitive test scores were compared across tertiles of the dietary contribution of UPF (%kcal UPF) using linear regression models. Further to unadjusted models (Model 1), three adjusted models were tested: (1) Model 2: adjusted for demographics (age, gender, ethnicity, education, and poverty–income ratio); (2) Model 3: demographics and lifestyle factors (physical activity, smoking status); (3) Model 4: demographics, lifestyle factors, BMI, and chronic diseases (history CVD, diabetes, and depression). Tests of linear trend were carried out by treating tertiles as a continuous ordinal variable. To examine potential differences in the association between UPF and cognitive scores by gender, age, BMI, diabetes, and history of CVD, Wald F tests were used to evaluate interaction terms in the fully adjusted model (Model 4). We tested the interaction with history of diabetes and CVD because individuals with chronic diseases are commonly recommended to improve their diet quality, which could result in reverse causality and bias results towards the null. Thus, we further performed the regression models among individuals with and without chronic conditions (history of CVD or diabetes) separately.

Sample weights provided by NHANES for the first day 24-h recall were used to account for the complex NHANES survey design including nonresponse and oversampling. Statistical analysis was performed with STATA/SE 16.0 for Windows (StataCorp LLC). Statistical hypotheses were tested using a two-tailed *P* ≤ 0.05 level of significance.

## Results

The average age of this study population was of 69 years, and 54% were females. The mean %kcal from UPF was 53%, ranging from 32.6% in the first tertile of UPF consumption (T1) to 70.4% in the third tertile (T3). No significant differences for age, gender, or poverty–income ratio were observed across the three UPF intake tertiles, but a lower percentage of other/multiracial and a higher percentage of non-Hispanic White and Black was observed in T3. Furthermore, we observed that individuals in T1 presented higher education attainment, as demonstrated by the higher percentage of participants who completed college. Overall, 72% of this study sample presented overweight or obesity (BMI ≥ 25 kg/m^2^). We observed an increasing trend in BMI and the prevalence of diabetes across the tertiles, while no significant differences were observed for physical activity, history of CVD, or depression (Table 1).

**Table 1:** Characteristics and measured data from NHANES 2011–2014 participants aged 60 years and over by dietary UPF tertiles (*n* = 2713)

Characteristics	All participants (*n* = 2713)	Dietary UPF tertiles (% of total energy intake)			*P* value
		T1 (0.7–43.9%)	T2 (43.9–60.1%)	T3 (60.2–100%)	
Age (years)^a^	69.1 (0.2)	69.0 (0.3)	69.5 (0.4)	68.8 (0.4)	0.581
Female, % (SE)^b^	53.9 (0.9)	55.3 (2.5)	52.5 (2.1)	54.0 (1.9)	0.693
Race/ethnicity, % (SE)^b^					< 0.001
Mexican American	3.5 (0.8)	3.3 (0.7)	4.4 (1.1)	2.9 (0.7)	
Non-Hispanic White	78.9 (1.9)	70.1 (2.4)	81.5 (2.3)	83.5 (2.1)	
Non-Hispanic Black	8.5 (1.2)	8.4 (1.3)	7.7 (1.2)	9.4 (1.7)	
Other/multiracial	9.1 (0.8)	18.2 (1.8)	6.4 (0.9)	4.2 (0.8)	
Missing	0	0	0	0	
Education, % (SE)^b^					0.002
Incomplete high school	15.8 (1.5)	15.0 (1.8)	14.4 (1.6)	17.8 (2.4)	
High school graduate	22.3 (1.4)	18.3 (1.6)	21.2 (2.1)	26.8 (2.3)	
Incomplete college	31.4 (1.4)	30.2 (2.9)	29.9 (2.1)	33.7 (1.9)	
College graduate	30.5 (2.0)	36.5 (3.3)	34.5 (2.8)	21.7 (2.1)	
Missing	0	0	0	0	
Poverty-income ratio, % (SE)^b^					0.738
< 1.30	16.4 (1.4)	15.7 (1.7)	16.2 (2.0)	17.2 (1.6)	
≥ 1.30	77.8 (1.3)	78.1 (2.1)	77.3 (2.5)	78.0 (1.8)	
Missing	5.8 (0.8)	6.2 (1.3)	6.5 (1.6)	4.8 (0.9)	
BMI (kg/m^2^)^a^	29.0 (0.2)	28.2 (0.3)	28.6 (0.3)	30.1 (0.4)	0.004
BMI status, % (SE)^b^					0.002
Underweight (< 18.5 kg/m^2^)	1.4 (0.3)	1.6 (0.6)	1.0 (0.4)	1.5 (0.5)	
Normal weight (18.5–24.9 kg/m^2^)	25.0 (1.3)	31.0 (2.1)	24.8 (2.1)	20.4 (2.0)	
Overweight (25–29.9 kg/m^2^)	35.2 (1.2)	33.7 (2.0)	39.6 (2.2)	32.4 (1.7)	
Obese (≥ 30 kg/m^2^)	37.1 (1.4)	32.6 (2.3)	33.7 (2.1)	44.2 (2.1)	
Missing	1.1 (0.3)	1.1 (0.4)	0.8 (0.3)	1.5 (0.6)	
Current smoker, % (SE)^b^					
Yes	10.6 (0.6)	10.3 (1.1)	8.7 (1.1)	12.7 (1.4)	0.110
Missing	0.04 (0.7)	0.004 (0.04)	0	0.09 (0.09)	
Physical activity (*z* score, min/day)^a^	0.08 (0.03)	0.17 (0.06)	0.09 (0.04)	0.01 (0.05)	0.093
History of CVD, % (SE)^b^					
Yes	79.6 (1.2)	76.2 (2.3)	79.1 (2.2)	82.9 (2.0)	0.105
Missing	0.08 (0.03)	0.16 (0.10)	0	0.09 (0.07)	
Diabetes, % (SE)^b^					
Yes	22.3 (0.9)	19.0 (1.2)	22.1 (1.8)	25.1 (1.7)	0.048
Missing	1.6 (0.4)	1.22 (0.3)	1.20 (0.5)	2.45 (0.8)	
Depression, % (SE)^b^					
Yes	7.0 (0.7)	6.8 (0.8)	5.5 (1.0)	8.5 (1.4)	0.063
Missing	0.8 (0.2)	0.4 (0.2)	0.6 (0.2)	1.3 (0.6)	
CERAD total score^a^	19.8 (0.2)	19.9 (0.2)	19.9 (0.3)	19.6 (0.3)	0.315
CERAD delayed recall^a^	6.3 (0.1)	6.3 (0.1)	6.2 (0.1)	6.3 (0.1)	0.964
Animal fluency^a^	18.1 (0.2)	18.2 (0.3)	18.3 (0.2)	17.9 (0.3)	0.301
DSST^a^	52.3 (0.6)	52.3 (0.8)	52.8 (0.9)	51.7 (0.9)	0.654
Dietary %kcal UPF^a^	53.0 (0.5)	32.6 (0.4)	52.4 (0.2)	70.4 (0.4)	–

Values presented as mean (standard error), unless stated otherwise (as % (SE))

*UPF* ultra-processed food

^a^Comparison between tertiles performed with linear regression

^b^Comparison between tertiles performed with Pearson chi-square

**Fig. 1:**
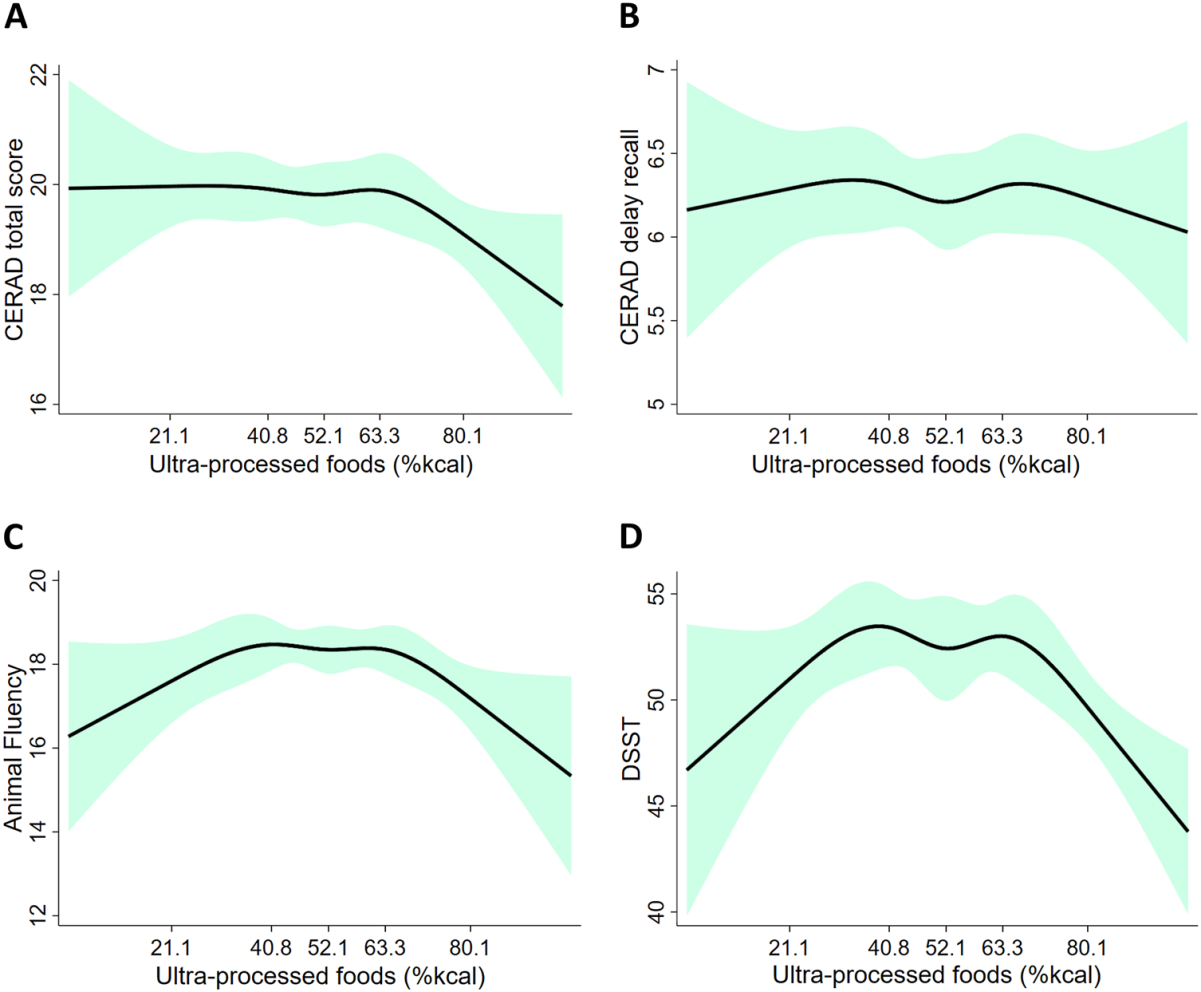
The relationship between dietary UPF (as % of total energy intake) and cognitive test scores: **a** CERAD total (*P* = 0.308); **b** CERAD delayed recall *(P* = 0.859); **c** animal fluency (*P* = 0.010); **d** digit Symbol Substitution test (DSST) (*P* = 0.005). Green area represents the 95% confidence intervals. *UPF* ultra-processed food, *%kcal* % of total energy intake

The graphical relationship between UPF and the cognitive test scores, assessed with restricted cubic spline plots, revealed an inverted U-shape association between UPF consumption and Animal fluency and DSST (Fig. 1). Thus, the association between performance in cognitive tests and UPF intake was assessed using tertiles of UPF.

No significant associations were observed between the UPF intake tertiles and the cognitive test results in the unadjusted and adjusted models (Table 2).

**Table 2:** Association between dietary UPF (as tertiles of % of total energy intake) and cognitive performance in adults ≥ 60 years old from NHANES 2011–2014 (*n* = 2713)

	Model 1		Model 2		Model 3		Model 4	
	*β* (95% CI)	*P* value	*β* (95% CI)	*P* value	*β* (95% CI)	*P* value	*β* (95% CI)	*P* value
CERAD recall								
T1 (Reference)	–	–	–	–	–	–	–	–
T2	− 0.07 (− 0.61, 0.46)	0.786	− 0.05 (− 0.48, 0.39)	0.823	− 0.05 (− 0.49, 0.39)	0.818	− 0.04 (− 0.50, 0.40)	0.857
T3	− 0.38 (− 0.93, 0.16)	0.162	− 0.35 (− 0.87, 0.16)	0.170	− 0.35 (− 0.87, 0.16)	0.167	− 0.32 (− 0.85, 0.19)	0.212
*P* for-trend		0.151		0.168		0.165		0.209
CERAD delayed recall								
T1 (Reference)	–	–	–	–	–	–	–	–
T2	− 0.04 (− 0.37, 0.29)	0.808	0.01 (− 0.26, 0.28)	0.934	0.01 (− 0.26, 0.28)	0.925	0.02 (− 0.25, 0.29)	0.888
T3	− 0.03 (− 0.31, 0.25)	0.815	0.01 (− 0.29, 0.31)	0.948	0.02 (− 0.28, 0.32)	0.914	0.04 (− 0.26, 0.33)	0.799
*P* for-trend		0.822		0.951		0.917		0.801
Animal fluency								
T1 (Reference)	–	–	–	–	–	–	–	–
T2	0.17 (− 0.67, 1.01)	0.681	0.00 (− 0.64, 0.64)	0.997	0.03 (− 0.59, 0.65)	0.920	0.04 (− 0.56, 0.65)	0.887
T3	− 0.30 (− 1.08, 0.47)	0.434	− 0.24 (− 0.83, 0.35)	0.405	− 0.19 (− 0.76, 0.38)	0.507	− 0.15 (− 0.69, 0.39)	0.583
*P* for-trend		0.389		0.383		0.483		0.560
Digit symbol								
T1 (Reference)	–	–	–	–	–	–	–	–
T2	0.52 (− 1.94, 2.99)	0.667	0.39 (− 1.14, 1.93)	0.603	0.43 (− 1.08, 1.94)	0.566	0.56 (− 0.98, 2.1)	0.464
T3	− 0.57 (− 2.57, 1.44)	0.569	− 0.20 (− 1.73, 1.32)	0.778	− 0.08 (− 1.60, 1.45)	0.918	0.29 (− 1.2, 1.8)	0.700
*P* for-trend		0.529		0.750		0.881		0.727

Model 1: unadjusted

Model 2: demographics (age, gender, ethnicity, education, and poverty–income ratio)

Model 3: demographics, lifestyle factors (physical activity and smoking status)

Model 4: demographics, lifestyle factor, BMI, and chronic diseases (history CVD, diabetes, and depression)

*UPF* ultra-processed food

Fully adjusted models were performed including interaction terms between UPF tertiles and gender, age, BMI, diabetes, and history of CVD. While no interactions were identified for gender, age, BMI, and CVD, the presence of diabetes interacted with UPF intake on DSST (Supplementary Table 1). That considered along with the possibility of reverse causality, where individuals with chronic diseases such as CVD and diabetes would change their dietary habits in response to health concerns, which could bias the analysis, we further performed the regression models among individuals with and without chronic conditions (history of CVD or diabetes) separately (Table 3). We observed a significant inverse association between the UPF intake tertiles and Animal Fluency test score, particularly in T3, in individuals without pre-existing chronic health conditions in all regression models, while no significant associations were observed for those with pre-existing chronic health conditions.

**Table 3:** Associations between dietary UPF (as tertiles of % of total energy intake) and cognitive performance in adults ≥ 60 years old with and without pre-existing chronic conditions (CVD or diabetes) from NHANES 2011–2014

	Model 1		Model 2		Model 3		Model 4	
	*β* (95% CI)	*P* value	*β* (95% CI)	*P* value	*β* (95% CI)	*P* value	*β* (95% CI)	*P*-value
Without pre-existing chronic health conditions (*n* = 453)								
CERAD recall								
T1 (Reference)	–	–	–	–	–	–	–	–
T2	− 0.27 (− 1.51, 0.97)	0.665	− 0.26 (− 1.33, 0.80)	0.620	− 0.32 (− 1.38, 0.74)	0.547	− 0.33 (− 1.35, 0.70)	0.523
T3	− 1.13 (− 2.52, 0.25)	0.106	− 0.72 (− 1.92, 0.47)	0.225	− 0.79 (− 2.02, 0.43)	0.195	− 0.75 (− 1.97, 0.50)	0.218
* P* for-trend		0.107		0.228		0.196		0.219
CERAD delayed recall								
T1 (Reference)	–	–	–	–	–	–	–	–
T2	0.09 (− 0.55, 0.74)	0.771	0.14 (− 0.43, 0.71)	0.620	0.15 (− 0.43, 0.72)	0.605	0.15 (− 0.41, 0.71)	0.591
T3	− 0.33 (− 1.10, 0.43)	0.380	− 0.08 (− 0.77, 0.61)	0.810	− 0.05 (− 0.75, 0.64)	0.872	0.01 (− 0.66, 0.68)	0.979
*P* for-trend		0.395		0.826		0.880		0.970
Animal fluency								
T1 (Reference)	–	–	–	–	–	–	–	–
T2	− 0.18 (− 2.12, 1.76)	0.852	− 0.21 (− 1.89, 1.47)	0.797	− 0.10 (− 1.76, 1.55)	0.898	− 0.09 (− 1.72, 1.54)	0.912
T3	− 2.04 (− 3.85, − 0.22)	0.029	− 1.53 (− 2.96, − 0.09)	0.037	− 1.40 (− 2.72, − 0.07)	0.040	− 1.37 (− 2.72, − 0.03)	0.046
*P* for-trend		0.030		0.040		0.041		0.049
Digit symbol								
T1 (Reference)	–	–	–	–	–	–	–	–
T2	− 1.80 (− 5.89, 2.89)	0.377	− 1.34 (− 4.37, 1.69)	0.375	− 1.51 (− 4.33, 1.31)	0.285	− 1.39 (− 4.14, 1.36)	0.312
T3	− 3.78 (− 9.04, 1.48)	0.153	− 1.35 (− 4.97, 2.27)	0.454	− 1.57 (− 5.07, 1.93)	0.368	− 1.27 (− 4.69, 2.15)	0.456
*P* for-trend		0.150		0.445		0.364		0.448
With pre-existing chronic health conditions (n = 2242)								
CERAD recall								
T1 (Reference)	–	–	–	–	–	–	–	–
T2	0.02 (− 0.62, 0.66)	0.948	− 0.01 (− 0.57, 0.54)	0.962	− 0.01 (− 0.57, 0.54)	0.955	− 0.02 (− 0.59, 0.54)	0.932
T3	− 0.11 (− 0.65, 0.42)	0.674	− 0.24 (− 0.75, 0.27)	0.347	− 0.24 (− 0.74, 0.28)	0.352	− 0.24 (− 0.76, 0.27)	0.340
*P* for-trend		0.648		0.337		0.343		0.331
CERAD delayed recall								
T1 (Reference)	–	–	–	–	–	–	–	–
T2	− 0.04 (− 0.40, 0.32)	0.811	− 0.01 (− 0.33, 0.30)	0.922	− 0.02 (− 0.33, 0.29)	0.906	− 0.02 (− 0.34, 0.29)	0.891
T3	− 0.08 (− 0.15, 0.31)	0.492	0.05 (− 0.24, 0.33)	0.735	0.05 (− 0.24, 0.33)	0.730	0.04 (− 0.23, 0.32)	0.748
*P* for-trend		0.446		0.718		0.712		0.728
Animal fluency								
T1 (Reference)	–	–	–	–	–	–	–	–
T2	0.42 (− 0.56, 1.39)	0.389	0.17 (− 0.67, 1.02)	0.679	0.15 (− 0.69, 0.99)	0.717	0.12 (− 0.72, 0.97)	0.764
T3	0.31 (− 0.54, 1.16)	0.465	0.15 (− 0.55, 0.85)	0.661	0.14 (− 0.53, 0.82)	0.667	0.13 (− 0.52, 0.78)	0.683
*P* for-trend		0.490		0.675		0.677		0.690
Digit symbol								
T1 (Reference)	–	–	–	–	–	–	–	–
T2	1.33 (− 1.37, 4.03)	0.323	0.75 (− 1.06, 2.57)	0.403	0.66 (− 1.14, 2.47)	0.457	0.65 (− 1.16, 2.45)	0.471
T3	0.74 (− 1.31, 2.79)	0.465	0.19 (− 1.51, 1.90)	0.818	0.21 (− 1.52, 1.95)	0.806	0.36 (− 1.39, 2.11)	0.681
*P* for-trend		0.519		0.880		0.857		0.721

Model 1: unadjusted

Model 2: demographics (age, gender, ethnicity, education, poverty–income ratio)

Model 3: demographics and lifestyle factors (physical activity, smoking status)

Model 4: demographics, lifestyle factors, BMI, and depression

*UPF* ultra-processed food

## Discussion

To our knowledge, this is the first study to investigate the association between cognitive performance and UPF consumption in older adults. While no significant associations between UPF intake and cognitive test scores were observed for this cross-sectional study in the US population, the consumption of UPF was inversely associated with performance in Animal Fluency, which assesses language and executive function, among those without pre-existing chronic health conditions such as CVD and diabetes.

Ageing is associated with structural and functional changes in the brain such as loss of synapses, decrease in neuronal plasticity and increase in oxidative stress and inflammation [34, 35]. As a consequence of these modifications, cognitive abilities are expected to decrease as part of normal ageing. Nonetheless, cumulative damage to the brain caused by a variety of factors may accelerate cognitive decline that results in a significant impairment and dementia. Modifiable factors, such as poor diet quality, diabetes, hypertension, and obesity, have been associated with decreased hippocampal size, which is then linked with a reduced capacity of neuroplasticity and increased risk of cognitive impairment [36, 37]. Given the influence of diet in modulating these risk factors, growing evidence shows that a healthy diet can have a positive impact in mitigating age-associated cognitive decline and prevent, or at least delay, dementia [38].

The consumption of UPF was inversely associated with performance in Animal Fluency among those without pre-existing chronic health conditions. Studies conducted in several countries, including the US revealed that UPF consumption is associated with poor diet quality characterised by high concentration of added sugars and saturated fatty acid [14], which are directly linked with cognitive decline and increased risk of dementia [39, 40].

Several biological mechanisms support the plausibility of our findings. Over the last decades, substantial evidence has linked Western diets, commonly rich in UPFs, high in fats and added sugars and low in fibers, with cognitive disfunction involving the hippocampus [37, 41]. Underlying mechanisms include systemic metabolic changes that lead to low-grade inflammation, impairment of the blood–brain barrier and neuroinflammation [39]. Furthermore, a link between UPF and cognitive decline relies on the increased risk for chronic diseases such as diabetes, CVD, and obesity, all known risk factors for cognitive impairment [2], among those with high UPF consumption [15, 17, 42]. Additionally, the gut–brain axis has been hypothesised to play a critical role on the association between UPF and cognitive impairment [23, 39, 41, 43]. Several neurobiological mechanisms may link high UPF consumption with gut microbiome alterations that potentially contribute to Western diets-mediated cognitive dysfunction, including reduced production of short-chain fatty acids, compromised barrier integrity, neuroinflammation, and peripheral and/or central insulin receptor resistance [39, 41]. This is likely due to the lack of essential nutrients and other bioactive compounds with anti-inflammatory and antioxidant properties and increased exposure to pro-inflammatory, toxic, and endocrine disruptors compounds (e.g., food additives and advanced glycation end products) in UPF diets [23, 43, 44].

This study took advantage of the high standards of the NHANES quality control on survey methods and data collection to examine the relationship between UPF intake and cognitive performance in older adults for the first time. However, potential limitations should be taken into consideration. CERAD Word Learning Test and DSST are known to have a ceiling effect, which should be considered when interpreting the results [45]. Nonetheless, in this study population, no one has reached the highest score on DSST (highest score = 105); furthermore, the maximum points were scored by only 3 participants in the CERAD recall, and 123 participants (4.5% of the total population) in the CERAD delayed recall.

Even though 24-h recalls are the least-biased self-report instrument available [46] and the standardised methods of NHANES have been shown to produce accurate intake estimates [47–49], dietary data obtained by 24-h recalls have some limitations [50]. This is especially true for UPF estimates given that information indicative of food processing level (i.e., place of meals, product brands) is not consistently determined for all food items and may not provide updated, market representative nutrient information [51]. Although the resultant misclassification could potentially over or underestimate the dietary contribution of UPF, such tendency is likely to be non-differential, biasing the association between UPF and cognition towards the null [52]. Should social desirability bias take people with obesity or chronic diseases to underreport consumption of foods with caloric sweeteners [53] such as desserts and sweet baked goods [54, 55], the dilution of the association between UPF consumption and cognition might be expected. On the other hand, residual confounding could overestimate the strength of the studied association particularly because common lifestyle risk factors tend to cluster [56] and higher levels of UPF consumption could be a proxy of an overall unhealthy diet or lifestyle. Furthermore, foods reported on the assessed days may not represent the usual diet, which may limit the precision of this method and bias the estimated associations between diet and health outcomes often towards the null. The cross-sectional nature of the study does not allow inferring causality. This is especially true if we consider that impaired cognition likely develops over years and may not be fully reversible. In this sense, reverse causality could dilute the studied association should people change their diet and consume less UPF after impaired cognition diagnosis. The null association between UPF consumption and Animal Fluency among participants with chronic conditions further speaks in this regard.

## Conclusions

Consumption of UPF was associated with worse performance in Animal Fluency, a cognitive test that assesses language and executive function in older adults without pre-existing diseases such as CVD and diabetes, while no associations were observed for those with these conditions. While longitudinal studies are required to provide stronger evidence, these results suggest that decreasing UPF consumption may be a way to mitigate age-associated cognitive decline and reduce the risk of dementia.

## Supplementary Information

Below is the link to the electronic supplementary material.

Supplementary file1 (DOCX 33 KB)

Supplementary file2 (DOCX 43 KB)

## Abbreviations

BMI: Body mass index
CAPI: Computer-assisted personal interviewing
CERAD: Consortium to establish a registry for Alzheimer’s disease
CVD: Cardiovascular disease
DASH: Dietary approaches to stop hypertension
DSST: Digit symbol substitution test
Hb1Ac: Glycohaemoglobin
MedDiet: Mediterranean diet
MIND diet: Mediterranean-DASH intervention for neurodegenerative delay diet
NCHS: National center for health statistics
NHANES: National health and nutrition examination survey
SD: Standard deviation
UPF: Ultra-processed food
USDA: United States Department of Agriculture

## Declarations

### Availability of data and materials

The datasets analyzed during the current study are available in the NHANES repository (https://wwwn.cdc.gov/nchs/nhanes/).
