# Supplementary material for: Association between ultra-processed food consumption and cognitive performance in US older adults: a cross-sectional analysis of the NHANES 2011–2014
Source: Eur J Nutr. 2022 Jul 1;61(8):3975–85. doi: 10.1007/s00394-022-02911-1 (PMC9596521; doi:10.1007/s00394-022-02911-1)
Supplement: Supplementary file 1 — Supplementary file1 (DOCX 33 KB) [file 394_2022_2911_MOESM1_ESM.docx]

**Supplementary Table S1.** Effect modification of gender, age, diabetes and CVD on the association between tertiles of ultra-processed food consumption and cognitive performance in NHANES 2011–2014 participants aged 60 years and over.

|  | **UPF intake × Gender** | **UPF intake × Age** | **UPF intake × BMI** | **UPF intake × Diabetes** | **UPF intake × CVD** |
| --- | --- | --- | --- | --- | --- |
| **Models^1^** | ***P*-value** | ***P*-value** | ***P*-value** | ***P*-value** | ***P*-value** |
| CERAD recall | 0.311 | 0.983 | 0.216 | 0.388 | 0.317 |
| CERAD delayed recall | 0.358 | 0.571 | 0.172 | 0.134 | 0.050 |
| Animal fluency | 0.162 | 0.749 | 0.573 | 0.497 | 0.134 |
| Digit symbol | 0.183 | 0.947 | 0.137 | **0.044** | 0.175 |

^1^ Wald F test. Models were adjusted for demographics (age, sex, ethnicity, education, poverty-to-income ratio), lifestyle factors (physical activity, smoking status), BMI and chronic diseases (history CVD, diabetes, depression). For each model, UPF intake, the covariate and their interaction were included.
