# Supplementary material for: Association between ultra-processed food consumption and cognitive performance in US older adults: a cross-sectional analysis of the NHANES 2011–2014
Source: Eur J Nutr. 2022 Jul 1;61(8):3975–85. doi: 10.1007/s00394-022-02911-1 (PMC9596521; doi:10.1007/s00394-022-02911-1)
Supplement: Supplementary file 2 — Supplementary file2 (DOCX 43 KB) [file 394_2022_2911_MOESM2_ESM.docx]

Supplementary Material

**Suppl Figure 1.** Selection of the study sample, from the National Health and Nutrition Examination Survey (NHANES) from 2011 to 2014.
